# Revascularization and Medical Therapy for Chronic Coronary Syndromes: Lessons Learnt from Recent Trials, a Literature Review

**DOI:** 10.3390/jcm12082833

**Published:** 2023-04-12

**Authors:** Vincent Pham, Alice Moroni, Emmanuel Gall, Alice Benedetti, Carlo Zivelonghi, Fabien Picard

**Affiliations:** 1 Department of Cardiology, Cochin Hospital, Hôpitaux Universitaire Paris Centre, Assistance Publique des Hôpitaux de Paris, 27 Rue du Faubourg Saint-Jacques, 75014 Paris, France; 2 Department of Cardiology, HartCentrum, Ziekenhuis Netwerk Antwerpen (ZNA) Middelheim, 2020 Antwerp, Belgium; 3 Faculté de Santé, Université Paris-Cité, 75006 Paris, France

**Keywords:** chronic coronary syndromes, CCS, revascularization, coronary artery disease

## Abstract

Stable coronary artery disease (CAD) has recently been replaced by a new entity described as chronic coronary syndrome (CCS). This new entity has been developed based on a better understanding of the pathogenesis, the clinical characteristics, and the morbi-mortality associated to this condition as part of the dynamic spectrum of CAD. This has significant implications in the clinical management of CCS patients, that ranges from lifestyle adaptation, medical therapy targeting all the elements contributing to CAD progression (i.e., platelet aggregation, coagulation, dyslipidaemia, and systemic inflammation), to invasive strategies (i.e., revascularization). CCS is the most frequent presentation of coronary artery disease which is the first cardiovascular disease worldwide. Medical therapy is the first line therapy for these patients; however, revascularization and especially percutaneous coronary intervention remains beneficial for some of them. European and American guidelines on myocardial revascularization were released in 2018 and 2021, respectively. These guidelines provide different scenarios to help physicians choose the optimal therapy for CCS patients. Recently, several trials focusing on CCS patients have been published. We sought to synthetize the place of revascularization in CCS patients according to the latest guidelines, the lessons learnt from recent trials on revascularization and medical therapy, and future perspectives.

## 1. Introduction

Coronary artery disease (CAD) is the first cardiovascular disease worldwide and is among the first causes of mortality. Chronic coronary syndromes (CCSs) have been defined to better characterize patients with CAD. This definition ought to replace the term of stable angina or stable ischemic heart disease. On the opposite of acute coronary syndromes (ACS), defined as unstable phases due to atherothrombotic events, CCS includes all the other phases of the history of coronary artery disease. The 2019 Guidelines of the European Society of Cardiology highlighted different clinical scenarios for CCS and provided guidelines regarding medical therapy and revascularization [1]. Medical therapy is the keystone in the management of patients with CCS, in order to improve both symptoms and prognosis. Revascularization, either with coronary artery bypass surgery (CABG) or percutaneous coronary intervention (PCI), should be considered on top of optimal medical therapy (OMT) in patients with a left ventricular ejection fraction (LVEF) ≤35% due to CAD, in case of multivessel disease, severe stenosis (>90% diameter), persistent angina, or large ischemia.

However, since the release of the latest guidelines regarding CCS, a large variety of trials have been published focusing on medical therapy and coronary revascularization on top of OMT. We sought to provide an update on lessons learnt from these recent trials.

## 2. Current Guidelines on Revascularization for Chronic Coronary Syndromes

**Figure 1:**
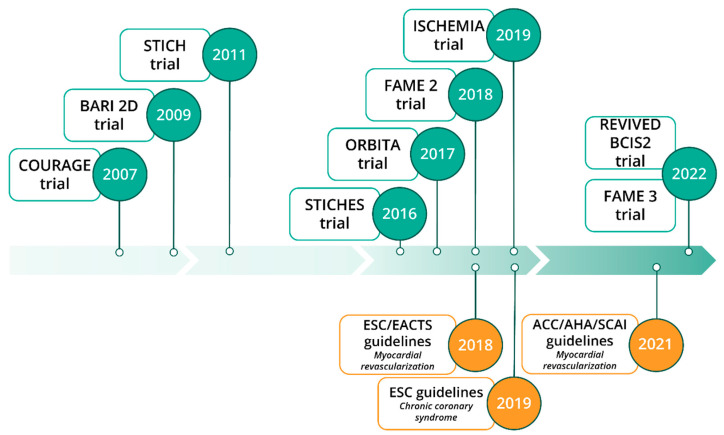
Impactful randomized clinical trials on revascularization in chronic coronary syndrome and current clinical guidelines. Abbreviations: ACC, American College of Cardiology; AHA, American Heart Association; BARI 2D, Bypass Angioplasty Revascularization Investigation 2 Diabetes; COURAGE, Clinical Outcomes Utilizing Revascularization and Aggressive Drug Evaluation; EACTS, European Association for Cardio-Thoracic Surgery; ESC, European Society of Cardiology; FAME, Fractional Flow Reserve vs. Angiography for Multivessel Evaluation; ISCHEMIA, International Study of Comparative Health Effectiveness With Medical and Invasive Approaches; ORBITA, Objective Randomised Blinded Investigation With Optimal Medical Therapy of Angioplasty in Stable Angina; REVIVED BCIS2, Revascularization for Ischemic Ventricular Dysfunction; SCAI, Society for Cardiovascular Angiography and Interventions, STICH, Surgical Treatment for Ischemic Heart Failure; STICHES, Surgical Treatment for Ischemic Heart Failure Extension Study.

The most recent European guidelines on myocardial revascularization were published in 2018 [2]. These guidelines were followed by specific ones focusing on CCSs in 2019. Then, in 2021, a report of the American College of Cardiology/American Heart Association Joint Committee [1,3] on myocardial revascularization was released (Figure 1). The 2019 European guidelines focused specifically on CCS while the American guidelines provided recommendations for the management of both ACS and stable ischemic heart diseases. Clinical trials have promoted the use of medical therapy as first-line management for CCS. Revascularization, although largely employed, should remain a therapeutic choice restricted to particular subgroups of CCS patients. The principal indications for revascularization in CCS are symptom relief in case of angina despite OMT and prognosis improvement by lowering cardiovascular (CV) events. CCS symptoms such as chest discomfort, pain or dyspnoea, are thought to be associated with myocardial ischaemia secondary to coronary stenosis. The general concept promoting the use of revascularization is that by restoring coronary blood flow, the burden of myocardial ischaemia is reduced leading to an improvement in angina and lower CV event rates.

### 2.1. Revascularization vs. Optimal Medical Therapy for Symptoms Relief

OMT represents the cornerstone of CCS management to reduce symptoms, delay disease progression, and prevent acute coronary events. In symptomatic patients, despite OMT, revascularization (either using PCI or CABG) has proven its effectiveness to reduce symptoms compared to OMT alone. More than a decade ago, the COURAGE (Clinical Outcomes Utilizing Revascularization and Aggressive Drug Evaluation) trial, showed a benefit of both medical therapy and PCI in improving the quality of life and symptoms relief. During the first 6 to 24 months, an additional benefit from PCI was observed; however, it disappeared at 36 months. Patients with most severe angina were the ones to benefit the most from PCI [4]. More recently, the ORBITA (Objective Randomized Blinded Investigation with optimal medical Therapy or Angioplasty in stable angina) trial compared the effects of PCI and OMT on exercise capacity and included a sham procedure [5]. The results found no clinical benefit from PCI compared to OMT and highlighted an important placebo component from PCI in symptoms relief. However, these results should be interpreted carefully due to the limited size of the study, which was stopped prematurely because of slow enrolment.

In the FAME 2 trial, a Fractional flow reserve (FFR)-guided PCI resulted in fewer patients with angina class II to IV (Canadian Cardiovascular Society II) as compared to OMT alone [6]. The benefit from PCI was greater at 30 days (HR 0.36 95%IC (0.26; 0.49); *p* < 0.001) and was maintained at the 3-year follow up (HR 0.54 95%IC (0.33; 0.89); *p* < 0.014) but disappeared at 5 years (HR 0.72 95%IC (0.45; 1.18); *p* < 0.19). Both European and American guidelines put a grade 1 recommendation for revascularization in symptomatic patients with CCS on top of medical therapy.

### 2.2. Revascularization for Major Cardiac Events Prevention

In stable coronary disease, revascularization should be performed in the presence of a large ischemia burden. To date, no clinical trial has shown a benefit of PCI over OMT alone on survival. In the COURAGE trial, PCI added to OMT resulted in a higher reduction in ischemia compared with OMT alone. Yet, that study found no difference between PCI and OMT on death or nonfatal myocardial infarction (MI) occurrence [7]. Although a meta-analysis including one hundred trials identified a modest effect on survival of revascularization with CABG and PCI using a drug-eluting stent, some recent data found no effect of PCI on mortality, cardiac death, or MI [8,9].

Nevertheless, the FAME 2 trial showed a significant reduction in urgent revascularization with an FFR-guided strategy compared to medical therapy alone. At a 5-year follow up, a strategy of FFR-guided PCI was associated with lower rates of a composite endpoint including death, MI, or urgent revascularization as compared to medical therapy (13.9% vs. 27.0%; *p* < 0.001) [6]. This difference was mainly driven by urgent revascularization (6.3% in the FFR group vs. 21.1% in the medical therapy group) and there was no significant difference on mortality rates or MI at 5 years.

In the case of a left main disease, data have shown a survival benefit of revascularization with CABG compared to medical treatment at a 5-year follow-up (10.2% vs. 15.8%; *p* = 0.0001). These results were consistent at a 10-year follow-up (26.4% vs. 30.5%; *p* = 0.03) [10]. Multiple studies have since found percutaneous coronary intervention to be a safe therapeutic option compared to surgery in unprotected left main stenosis in patients with low or intermediate anatomical coronary complexity [11].

The 2019 ESC guidelines [1], identifies different practical clinical scenarios to guide revascularization. Whether patients are symptomatic or not, revascularization should be considered in the following scenarios:

- Documented ischemia (ischemia should be >10% in asymptomatic patients);
- Diameter stenosis >90%;
- FFR < 0.80 or instantaneous wave-free ratio (iFR) <0.89 in major vessels;
- Left ventricular ejection fraction (LVEF) <35% due to coronary artery disease [1];
- The 2021 [3] American guidelines put the emphasis on the indication of revascularization in stable ischemic heart disease (SIHD), whether it is performed for symptoms relief (class of recommendation 1) or to improve prognosis. Revascularization with coronary artery bypass is recommended to improve prognosis in multivessel disease and left ventricular ejection dysfunction <35% (class of recommendation 1) and <50% (class of recommendation 2a) or in case of significant left main stenosis. If the anatomy is suitable, PCI is recommended for left main stenosis (class of recommendation 2a). However, in case of SIHD and a normal left ejection fraction, the interest of revascularization on survival is uncertain in multivessel disease and proximal left anterior descending stenosis (class of recommendation 2b).

## 3. Recent Trials on Revascularization vs. Optimal Medical Therapy in Chronic Coronary Syndromes

### 3.1. PCI vs. OMT: Lessons from ISCHEMIA Trial

Among patients with stable coronary disease, COURAGE and BARI 2D (Bypass Angioplasty Revascularization Investigation 2 Diabetes) trials [4,12] failed to demonstrate any significant benefit from coronary revascularization compared to OMT in the occurrence of all-cause death or CV outcomes. One of the criticisms of all these trials was the possibility that all patients with a most severe CAD involvement that might be associated with a very high risk for adverse ischemic outcomes were likely not randomized but sent directly to invasive revascularization. To avoid this major selection bias, it was essential to randomize patients before coronary angiogram. This is what led to the ISCHEMIA trial [13] promoted by the American National Institutes of Health. In line with historical trials, the ISCHEMIA trial [13] did not find evidence that an initial invasive strategy, as compared with an initial conservative strategy, reduced the risk of ischemic CV events or death from any cause over a median of 3.2 years in 5179 patients with documented moderate or severe ischemia on stress tests. Despite important strengths related to trial design with a very low rate of patients lost to follow-up (<1%), there were also several limitations to note. First, the statistical power was decreased by reducing the sample size from 8000 to 5179 patients and event rates was lower than expected. Second, the trial findings were sensitive to the definition of MI that was used, and a subanalysis that excluded procedural infarctions suggested that initial coronary revascularization could improve outcomes at 5 years of follow-up [13]. The extended follow-up of the ISCHEMIA trial [13] will provide more information since survival curves have crossed through the study period. Third, most patients included were asymptomatic or only mildly symptomatic at baseline, when it is precisely the effect of revascularization on effective angina relief that is expected. These findings suggest that OMT in asymptomatic or mildly symptomatic patients might be the best initial strategy without the benefit of coronary revascularization, whereas invasive strategy would be a reasonable complementary approach for symptomatic patients with frequent angina episodes. Furthermore, all participants were screened before randomization with a coronary computed tomography angiography (CCTA) and were excluded from the ISCHEMIA trial [13] in case of left main stenosis of at least 50%.

### 3.2. PCI vs. OMT in Heart Failure with Reduced-Ejection-Fraction Patients: Lessons from REVIVED-BCIS2 Trial

Coronary artery disease is the leading cause of heart failure. Since the STICHES trial [14], it is known that a strategy of coronary artery bypass grafting (CABG) added to guideline-directed medical therapy compared to medical therapy alone brings a benefit for 10-year survival. 

However, until now, we lacked data comparing PCI to medical treatment in this subset of patients. The REVIVED-BCIS2 trial [15] compared PCI to OMT (i.e., individually adjusted pharmacologic and device therapy for heart failure) in patients with heart failure with impaired left ventricular ejection fraction (LVEF) and advanced coronary disease with proven viability for at least four myocardial segments. The primary composite outcome was death from any cause or hospitalization for heart failure. Major secondary outcomes were LVEF at 6 and 12 months and quality-of-life scores. In total, 700 patients were randomized over a 7-year period into the two groups, with a mean follow-up of 41 months. The population was predominantly male (87%), with half of it with sequelae of MI, and a mean LVEF of 27%.

Over a median of 41 months, no difference could be observed between the PCI group and the OMT group with respect to the primary endpoint (37.2% vs. 38%; HR: 0.99; *p* = 0.96), mortality (31.7% vs. 32.6%; HR = 0.98), or hospitalization for heart failure (14.7% vs. 15.3%; HR = 0.97). The LVEF was similar in the two groups at 6 months (mean difference, −1.6 percentage points; 95% CI, −3.7 to 0.5) and at 12 months (mean difference, 0.9 percentage points; 95% CI, −1.7 to 3.4). Quality-of-life scores at 6 and 12 months appeared to favour the PCI group, but the difference had diminished at 24 months. Several limitations should be noted. First, the median follow-up was quite short while the benefit of myocardial revascularization was observed in the STICHES trial [14] over 10 years of follow-up. On the other hand, details about the coronary anatomy of the included population were not known. In addition, there was a higher rate of infarction and urgent revascularization in the medical treatment group, which could have a prognostic impact in the long term.

In addition to the long-term follow-up of participants, further prospective multicentre randomized clinical trials are warranted to investigate the optimal strategy between CABG and PCI for selected patients with severe ischemic cardiomyopathy.

### 3.3. FFR-Guided PCI vs. CABG for Three-Vessel Coronary Artery Disease: Lessons from FAME Trial

Patients with complex three-vessel coronary artery disease have been found to have better outcomes with CABG than with PCI [16,17], especially in the case of diabetes mellitus [18]. When coronary lesions are less extensive (defined by a SYNTAX score ≤22), PCI is recommended equally to CABG in nondiabetic patients. However, trials have rarely used second-generation drug-eluting stents (DES) and have not routinely measured FFR to guide PCI. FFR-guided PCI has led to more judicious stenting for only functionally significant coronary stenosis and is associated with a significant reduction in cardiovascular events [19]. In addition, second-generation DES have improved early and late outcomes, leading to lower rates of associated stent thrombosis, procedural and spontaneous MI, restenosis, and death than first-generation drug-eluting stents [20]. However, whether FFR-guided PCI performed with current-generation DES was noninferior to CABG on cardiovascular events was still unclear until the FAME 3 trial.

The FAME 3 trial [21] was a multicentre, international, noninferiority trial, which included 1500 patients with three-vessel coronary artery disease who were randomly assigned to undergo CABG or FFR-guided PCI. The primary endpoint was the occurrence within 1 year of a major adverse cardiac or cerebrovascular event, defined as death from any cause, MI, stroke, or repeat revascularization.

The patients included were relatively young (mean age of patients was 65.2 ± 8.6 years), with 29% of patients with diabetes, and presented a relatively complex coronary anatomy (mean SYNTAX score of 26). At the 1-year follow-up, FFR-guided PCI did not achieve noninferiority as compared with CABG regarding the composite primary endpoint (10.6% vs. 6.9%; CI, 1.1 to 2.2; *p* = 0.35). The incidence of the secondary composite endpoint of death, MI, or stroke and of each individual component of the primary endpoint did not differ significantly between the two groups. Incidences of procedural complications such as major bleeding, acute kidney injury, arrhythmia, and rehospitalization within 30 days were higher and the mean length of hospital stay longer among the patients randomly assigned to undergo CABG.

Despite the undoubted improvements offered by FFR in the settings of PCI, CABG with left internal artery thoracic artery used as grafts should be the preferred strategy in patients with complex and diffuse CAD. A further analysis on long-term follow-up at 3 and 5 years and information about the changes in quality of life and cost-effectiveness are expected.

## 4. Recent Trials on Medical Therapy Optimization in Chronic Coronary Syndromes

Medical therapy plays a pivotal role in preventing the progression of atherosclerotic disease and the recurrence of atherothrombotic events in patients with coronary artery disease (CAD) [22].

Recent literature has highlighted the crucial contribution of platelet aggregation, coagulation, cholesterol, and systemic inflammation to CAD progression [23], even though the pathobiology of recurrent events post-ACS may slightly differ from that of CCS patients who have not experienced a previous ischemic event.

**Figure 2:**
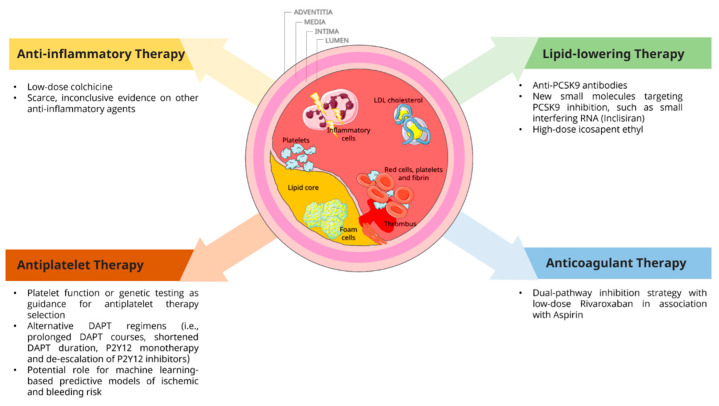
Therapeutic targets and medical treatment novelties in chronic coronary syndrome. Platelet aggregation, coagulation, cholesterol, and systemic inflammation all play a pivotal role in coronary artery disease progression and thus represent the targets for medical therapeutical interventions. Here represented are the recent advances in antiplatelet, anticoagulant, anti-inflammatory, and lipid-lowering medical therapy. Abbreviations: DAPT, dual anti-platelet therapy; LDL, low-density lipoprotein; PCSK9, proprotein convertase subtilisin kexin type 9.

The complexity of CAD progression poses a mechanistic basis for therapeutical interventions targeting the atherosclerotic burden as a whole [24] (Figure 2), thus emphasizing the importance of optimal medical management beyond (or in place of) invasive revascularization of flow-limiting/ ischemia-driving lesions.

### 4.1. Antiplatelet Therapy

Antiplatelet therapy is the cornerstone of pharmacological therapy in patients with CAD at elevated risk of ischemic events or with previous ischemic events to modulate the progression of atherosclerotic disease [25,26]. Due to a progressive refinement in ischemic risk stratification together with a deeper understanding of bleeding-associated prognostic implication, antiplatelet treatment regimens have substantially evolved over the last decade [27].

In patients undergoing PCI, dual antiplatelet therapy (DAPT) is usually the strategy of choice, as recommended by current guidelines [22]. Despite being the most widely used P2Y12 inhibitor agent, clopidogrel has shown substantial heterogeneity in individual response with inadequate platelet inhibitory effects, and could result in increased thrombotic event rates [28]. Therefore, some alternative strategies based on more potent P2Y12 inhibitors have been proposed and tested in randomized clinical trials (RCT). Nevertheless, RCTs conducted in high-risk CCS patients undergoing PCI did not demonstrate any short-term benefit of prasugrel and ticagrelor over clopidogrel [29,30]. However, a recent meta-analysis in patients with ACS or CCS undergoing PCI highlighted the efficacy and the favourable safety profile of platelet function or genetic testing as guidance for antiplatelet therapy selection compared with standard selection [31], and such strategy proved to be cost-effective [32,33,34]. These findings suggest a potential role for a guided approach to inform on P2Y12 inhibitors’ escalation to reduce thrombotic complications [35], in light of the wider availability of genetic testing in routine clinical practice [36].

In addition to this, the high risk for long-term ischemic recurrences in patients with CAD has driven research on prolonged DAPT duration. The results of the DAPT and Prevention of Cardiovascular Events in Patients With Prior Heart Attack Using Ticagrelor Compared to Placebo on a Background of Aspirin-Thrombolysis in Myocardial Infarction (PEGASUS-TIMI) 54 trials have endorsed the implementation of prolonged DAPT courses (beyond 12 months) in patients with ACS or CCS and an increased thrombotic risk [37,38]. In particular, ticagrelor 60 mg bis in die (b.i.d.) have emerged as a well-tolerated option even in patients with CCS and type II diabetes without a history of MI or stroke [39], especially in those treated with previous coronary stenting [40]. Nevertheless, a parallel significant increase in moderate and severe bleeding occurred in all prolonged DAPT combinations [41]. These considerations have prompted the development of tailored antiplatelet regimens balancing the ischemic and bleeding risk of the patient.

Indeed, by contrast, in case of occurrence of bleeding complications or concerns for potential bleeding, specific pharmacological strategies should be contemplated, namely a shortened DAPT duration, P2Y12 monotherapy, and a de-escalation of P2Y12 inhibitors. Indeed, unlike the ischemic risk that declines 1 to 3 months after PCI, the bleeding risk generally plateaus over time. A DAPT shortening was explored in multiple studies. The STOPDAPT-2 (a multicentre, open-label, adjudicator-blinded randomized clinical trial in Japan designed to compare 1 month of DAPT with 12 months of DAPT after drug-eluting stent implantation) showed that 1 month of DAPT followed by clopidogrel monotherapy, compared with 12 months of DAPT with aspirin and clopidogrel, resulted in a significantly lower rate of a composite of cardiovascular and bleeding events, meeting the criteria for both noninferiority and superiority [42]. In addition, the SMART-CHOICE [43], a multicentre, open-label, noninferiority, randomized study performed at 33 sites in Korea designed to compare 3 months of DAPT with 12 months of DAPT after drug-eluting stent implantation showed that P2Y12 inhibitor monotherapy after 3 months of DAPT compared with prolonged DAPT resulted in noninferior rates of major adverse cardiac and cerebrovascular events. A recent meta-analysis by Khan et al. [44] showed no significant differences in the risks of ischemic endpoints or major bleeding between mid- or short-term DAPT followed by aspirin monotherapy, in comparison with a 12-month DAPT. On the other hand, short-term DAPT followed by P2Y12 inhibitor monotherapy was associated with a reduced risk of major bleeding. There were no significant differences with respect to mortality between the different DAPT strategies. That meta-analysis suggested that a 1-month DAPT followed by P2Y12 inhibitor monotherapy was superior to a 3-month DAPT followed by aspirin monotherapy to limit bleeding risk. Nevertheless, although DAPT shortening was explored in numerous studies, only the Management of High Bleeding Risk Patients Post Bioresorbable Polymer Coated Stent Implantation with an Abbreviated vs. Standard DAPT Regimen (MASTER-DAPT) RCT selectively enrolled patients with a high bleeding risk [45]. This trial showed noninferiority for preventing adverse clinical events and superiority for limiting bleeding events of a 1-month DAPT after implantation of a biodegradable-polymer sirolimus-eluting stent in the context of ACS or CCS. Regarding P2Y12 monotherapy, a 1- or 3-month DAPT effectively reduced the hazard of any bleeding without increasing the risk of ischemic events, as demonstrated by many RCTs and corroborated by meta-analyses [27,46,47,48]. Nevertheless, early withdrawal of aspirin must be undertaken with caution in ACS patients when clopidogrel is used as monotherapy as a nearly twofold increase in the risk of MI was observed in the case of a 1- to 2-month DAPT followed by clopidogrel monotherapy [49]. Finally, a de-escalation of P2Y12 inhibitors consisting in a switch from more (prasugrel or ticagrelor) to less (clopidogrel) potent agents typically applies to the setting of ACS. Overall, a de-escalation of antiplatelet therapy among patients undergoing PCI is associated with a significant reduction of bleeding, without drawbacks in efficacy, irrespective of a guided (by use of genetic or platelet tests) or unguided strategy, as compared to the standard DAPT selection with potent P2Y12 inhibitors [49].

The recent implementation of machine-learning-based models to predict post-ACS ischemic and bleeding risks, overcoming the intrinsic limits of currently recommended scores, has the potential to provide new clues for tailored decision-making regarding the choice of optimal antithrombotic therapy [50].

### 4.2. Anticoagulant Therapy

A novel dual-pathway inhibition (DPI) strategy with low-dose (LD) factor-Xa inhibitor (rivaroxaban 2.5 b.i.d.) in association with aspirin 100 mg once daily (o.d.) has received approval as maintenance treatment beyond 12 months post-ACS PCI [51]. In the Cardiovascular OutcoMes for People using Anticoagulation StrategieS (COMPASS) trial, 27,395 subjects with CCS, peripheral arterial disease, or both (overall, 91% had CAD) were randomized to a regimen of LD-rivaroxaban plus aspirin, rivaroxaban 5 mg b.i.d., or aspirin alone [52]. The former combination reduced the primary outcome (a composite of MI, stroke, or cardiovascular death) by 26% and death by 24%, compared with treatment with aspirin alone. These results were achieved at the expense of a 69% increase in major bleeding, albeit without significant increase in either intracranial or fatal bleeding [53]. Of note, in a recent network meta-analysis, LD-rivaroxaban plus aspirin was the most preferable long-term antithrombotic regimen for CCS patients with high-risk factors, compared to ticagrelor plus aspirin, rivaroxaban monotherapy, and thienopyridine plus aspirin [41]. According to current guidelines, prolonged antithrombotic treatment with either LD-rivaroxaban or P2Y12 inhibitors in addition to aspirin should and may be considered in patients at high and moderately elevated thrombotic risk, respectively [51].

### 4.3. Lipid-Lowering Therapy

Beside statins and ezetimibe as a backbone of lipid-lowering therapy in primary and secondary prevention, new agents targeting proprotein convertase subtilisin kexin type 9 (PCSK9) have gained remarkable interest due to their potential for plaque modification ultimately leading to a significant reduction of major adverse cardiovascular events (MACE) [54]. The first anti-PCSK9 antibodies, alirocumab and evolocumab, were associated with a dramatic decrease in low-density lipoprotein-cholesterol (LDL-C) levels and MACE by approximately 60% and 20%, respectively [55,56]. Of note, in addition to directly intervening in lipid metabolism, lipid-lowering medication has shown pleiotropic beneficial effects on atherosclerotic plaque healing allowing for plaque stabilization, as assessed by in vivo optical coherence tomography (OCT) imaging studies [57,58,59]. Anti-PCSK9 antibodies have reached a class I recommendation in patients not achieving their LDL-C goals on a maximum tolerated dose of statin and ezetimibe in the secondary prevention setting, whereas they may be considered (class IIb recommendation) for primary prevention in patients at very high cardiovascular risk [60]. Among the new small molecules targeting PCSK9 inhibition [61], the small interfering RNA inclisiran has shown promising results in reducing LDL-C levels on top of maximum tolerated guideline-recommended statin treatment [62], finally reaching EMA approval in December 2020 for use in adults with primary or mixed dyslipidaemia [63]; the potential benefits of inclisiran on cardiovascular outcomes are currently being tested (ORION-4, NCT03705234). Regarding therapy targeting blood triglyceride levels, the recent Reduction of Cardiovascular Events With Icosapent Ethyl–Intervention Trial (REDUCE-IT) demonstrated the safety and effectiveness of high-dose icosapent ethyl on top of statin therapy in lowering triglycerides, with a significant reduction of CV events and CV death [64]. Of note, the study cohort was composed of patients with either known CV diseases or at high CV risk.

### 4.4. Anti-Inflammatory Therapy

Systemic inflammation has recently emerged as a potential new therapeutic target to reduce cardiovascular events. Statin therapy is known to reduce C-reactive protein (CRP) [65], a marker of inflammation; by contrast neither evolocumab nor alirocumab have shown significant effects on inflammatory markers in ACS patients.

Several RCTs have evaluated the effect of “direct” anti-inflammatory therapies, investigating whether intervening on inflammation independently of LDL-C lowering could confer a clinical benefit. Initially, interleukin-1β antagonist canakinumab (150 mg every 3 months) and low-dose colchicine (0.5 mg o.d.) proved to be beneficial in reducing CRP levels and MACE rate in patients with prior MI and persistent inflammatory residual risk [66,67]. However, canakinumab was not further developed in this setting because of the risk of fatal infections and the high costs, whereas the subsequent LoDoCo2 (second low-dose colchicine) trial and the two-year follow-up of the COPS trial expanded the positive results of colchicine in patients with CCS [68,69]. By contrast, the recent COVERT-MI trial failed to show a reduction in infarct size, microvascular obstruction, and LV adverse remodelling with a high-dose short-term colchicine administration (2 mg loading dose followed by 0.5 mg twice a day for 5 days) in the acute phase of ST-segment-elevation MI [70], thus posing the need for further studies exploring the timing, pharmacokinetics, and dose–response of colchicine.

Other studies on anti-inflammatory agents vs. placebo have been published but have failed to show remarkable improvements in MACE, likely because of their limited sample sizes. A recent meta-analysis (comprising 18 RCTs with a total of 67,449 participants) overcoming such limitations found a lower risk of recurrent MI in CCS patients treated with anti-inflammatory therapies, albeit at the cost of increased infection [71]. Colchicine was associated with the most favourable cardioprotective effect. Accordingly, low-dose colchicine is the only anti-inflammatory agent mentioned in the guidelines as possible adjunctive therapy in secondary prevention (class IIb recommendation) [72].

## 5. Unmet Needs and Future Perspectives of Percutaneous Myocardial Revascularization in Chronic Coronary Syndrome

Previous trials sought to evaluate the clinical effect of myocardial revascularization added to medical therapy in stable CAD. However, they could not prove any reduction in the incidence of death or myocardial infarction in patients treated with myocardial revascularization and optimal medical therapy compared to patients treated with optimal medical therapy alone [7,12]. As detailed above, the ISCHEMIA trial has recently demonstrated that an initial invasive strategy compared to an initial conservative strategy did not reduce the incidence of clinical events at follow-up [13]. This result was probably driven by the higher incidence of periprocedural MI in the invasive strategy group which balanced the higher incidence of late cardiovascular events in the conservative strategy group. Indeed, the conservative strategy group presented a higher rate of spontaneous MI than the invasive strategy group. Of note, spontaneous MI was previously demonstrated to determine a higher risk of mortality than periprocedural infarctions [73].

**Figure 3:**
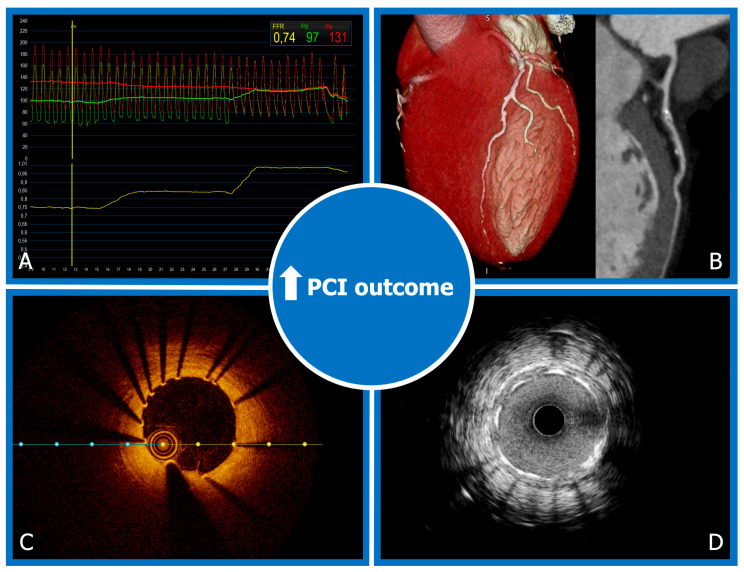
PCI optimization strategies to improve clinical outcome. Physiology assessment with FFR pullback (**A**). Coronary CTA showing anatomical 3D and multiplanar reconstructions (**B**). Post-PCI OCT and IVUS evaluation of stent expansion and apposition (**C**,**D**).

The persistence of a considerable incidence of periprocedural MI, which may affect the overall clinical benefit of PCI, have led in the last decades to the development of additional techniques for PCI optimization in order to limit periprocedural events and target vessel failure at follow-up. Moreover, advanced techniques of plaque characterization may contribute to recognizing which lesions present instability features that could determine a higher risk of spontaneous events in patients with stable CAD. In particular, invasive physiology assessment and intravascular imaging may help to identify which patients might obtain more clinical benefit from PCI and play an essential role in PCI optimization (Figure 3). Moreover, CCTA may provide an accurate characterization of a coronary lesion other than the possibility to plan PCI strategies. Finally, given the occurrence of in-stent restenosis, which also affects a coronary lesion treated with last-generation drug-eluting stent (DES), drug-coated balloon (DCB) utilization has been proposed in some particular settings to decrease the risk of target vessel failure.

### 5.1. Percutaneous Myocardial Revascularization and Coronary Physiology Assessment

The invasive physiological assessment of intermediate coronary artery stenosis with FFR has been shown to represent a useful method for functional severity evaluation [74]. The FAME and FAME II trials widely demonstrated the clinical benefits of FFR-guided PCI in patients with stable CAD compared to angiographically guided PCI and OMT alone, respectively [6,19,75]. With regard to FFR evaluation for PCI optimization, the FFR SEARCH and TARGET-FFR studies highlighted that more than half of patients presented a suboptimal post-PCI FFR (FFR ≤ 0.90) [76,77]. In addition, an inverse relationship between post-PCI FFR and clinical events was shown in a previous meta-analysis [78]. Other studies revealed that lower FFR values after PCI were associated with increased adverse clinical events [79] and higher post-PCI FFR values were related to a higher rate of angina symptoms relief and a lower number of clinical events [80]. One of the described mechanisms leading to lower post-PCI FFR values was the presence of residual diffuse coronary atherosclerosis, which may not be detected on coronary angiography [79,81].

The FFR assessment provides additional useful information when a pullback measurement is performed and recorded in the target vessel [74]. This allows for a discrimination between focal or diffuse CAD, which could correspond to two different pathophysiological and clinical entities. The basic hypothesis, currently under investigation, is that focal stenoses are associated with more pronounced vulnerable plaque features (because of turbulent flow and higher shear-stress forces) and thus more prone to instability and adverse clinical events. These lesions would likely benefit more from an invasive treatment. On the contrary, a diffuse atherosclerotic pattern consists more of “stabilized plaques”, with less lipidic pools and necrotic cores, thus suggesting a less aggressive condition. To characterize the atherosclerosis distribution in the coronary vessel, discriminating between focal and diffuse disease, and to quantify the magnitude of pressure loss along the vessel, the hyperaemic pullback pressure gradient (PPG) index was recently developed [82]. The authors proved that patients with a diffuse disease, defined by PPG values <0.66, presented a higher rate of post-PCI residual angina than patients with a focal disease (52% vs. 28%, respectively, *p* = 0.02) [83]. In addition, PCIs performed in the diffuse disease, defined by PPG, resulted in a smaller improvement of post-PCI FFR values than in the focal disease [84]. The PPG Global registry (NCT04789317) will provide more information about PPG cutoff values and its implications in stable CAD treatment. Moreover, further investigations with randomized clinical trials are expected to define the real benefit of PCI in patients with diffuse CAD compared to medical therapy alone. Additional ongoing research will evaluate the clinical impact and the management of focal coronary lesions characterized by high PPG values in presence of negative FFR values.

As far as non-hyperaemic physiological indexes are concerned, the SWEDEHART and DEFINE-FLAIR trials showed that iFR-guided revascularization was noninferior to FFR-guided revascularization [85,86,87]. The ongoing DEFINE GPS trial (NCT04451044) will provide further evidence about the clinical benefits of iFR-guided PCI comparing the clinical outcome of iFR-guided PCI with a coregistration system vs. angiographically guided PCI.

Other non-hyperaemic indexes such as the resting full-cycle ratio (RFR) have been validated in the last few years [88]; however, more data to evaluate their role in PCI guidance and optimization are needed.

### 5.2. Intravascular Imaging for High-Risk Plaque Characterization and PCI Optimization

Recent studies have emphasized the clinical impact of coronary plaque morphology assessment by optical coherence tomography (OCT) [89]. This intravascular imaging technique allows the characterization of the plaque composition highlighting instability features that could determine a high risk of rupture and CV events. In particular, the evidence of thin-cap fibroatheroma (TCFA) has been shown to be associated with a higher risk of clinical events determining plaque vulnerability [90,91]. The COMBINE OCT-FFR trial revealed that, in patients with diabetes mellitus and FFR-negative lesion, the evidence of TCFA was related to a higher incidence of MACE at the 18-month follow-up. In addition, the presence of TCFA represented the strongest predictor of adverse clinical events [92]. Given this result, OCT may play a significant role in the identification of patient affected by high-risk plaque who might benefit from PCI avoiding subsequent spontaneous cardiovascular events. However, further evidence is needed to assess whether a percutaneous treatment of TCFA, regardless of the presence of ischemia, may improve clinical outcomes. The ongoing PREVENT (NCT02316886) randomized trial promises to shed light on the clinical impact of PCI among patients with coronary lesions characterized by vulnerable plaques.

Regarding post-PCI imaging, minimal stent area (MSA) and stent expansion represent relevant predictors of PCI outcomes [93,94]. Indeed, stent underexpansion has been proven to be associated with a higher risk of target lesion failure including stent thrombosis [95,96,97]. Moreover, IVUS-guided PCI leads to larger luminal vessel dimensions compared to angiographically guided PCI and a lower incidence of MACE at the 1-year follow-up [98,99]. Despite its established clinical implications, a high rate of stent underexpansion has been shown in the latest studies, even when percutaneous revascularization was performed using IVUS or OCT. The ILUMIEN III: OPTIMIZE PCI trial described that 59% of cases treated with OCT-guided PCI and 63% of those treated with IVUS-guided PCI presented stent underexpansion (stent expansion <90%) [100]. In the ULTIMATE trial, 21% of the lesions treated with IVUS-guided PCI presented MSA less than 5 mm [2] and less than 90% of lumen cross-sectional area at the distal reference segments [101]. Finally, the IVUS-XPL trial demonstrated that 46% of patients treated with IVUS-guided PCI did not show a proper stent optimization in terms of stent expansion [98]. This high variability of suboptimal PCI rate seems related to different definitions of stent underexpansion adopted in these studies. Indeed, a standard definition of stent underexpansion is still lacking. It would be useful to understand from further investigations which index of stent expansion best predicts clinical outcomes in order to identify in which cases stent optimization has to be performed to avoid the occurrence of adverse clinical events.

The definition of stent malapposition is well established in both IVUS and OCT intravascular imaging evaluations [102]; however, its impact in determining stent failure is still debated. Large studies have not revealed any relationship between acute stent malapposition detected with IVUS [103] or OCT [100] and clinical outcomes. On the other hand, the presence of stent malapposition has been described as a frequent finding in the case of stent thrombosis but these studies could not differentiate between late-persistent stent malapposition and late-acquired stent malapposition [95,96]. In the research by Im E. et al., only 31% of lesions with acute stent malapposition remained malapposed at follow-up, while late-acquired stent malapposition was detected in 15% of cases, and no clinical events occurred during the follow-up in patients with late malapposition [104]. Larger studies evaluating clinical outcomes in patients with acute or late malapposition are needed to clarify their clinical impact especially in a long-term follow-up.

### 5.3. Role of Coronary Computed Tomography Angiography in Coronary Artery Disease

CCTA represents a noninvasive method for CAD evaluation [105]. In recent years, advanced methods for coronary flow measurement and coronary physiology assessment from CCTA images have been developed. FFR measurements derived from CCTA have showed a comparable accuracy to invasive physiology evaluation [106]. Moreover, it has been recently proven that FFR-CT is also accurate and precise for predicting FFR after PCI [107]. This functional analysis together with an extensive anatomical evaluation of the coronary tree may be involved in the PCI planning process of stable CAD. In particular, the anatomical visualization of coronary stenosis, the plaque composition characterization, and the assessment of calcium distribution may contribute to the decision of the best PCI strategy. In addition, the prediction of post-PCI FFR values may reveal the final FFR result of the revascularization strategy. The achievement of optimal PCI results through the application of a CCTA analysis may determinate the reduction of periprocedural and long-term cardiovascular events. The ongoing Precise Procedural and PCI Plan Randomized Clinical Trial Integration of Coronary Computed Tomography Angiography in the Catheterization Laboratory to Plan and Guide Coronary Percutaneous Procedures (P4) trial (NCT05253677) will explore the clinical benefit of CTA-guided PCI comparing clinical outcomes between patients treated with CTA-guided PCI and IVUS-guided PCI.

### 5.4. Latest Evidence on Drug-Coated Balloons in Percutaneous Coronary Treatment

DES implantation has been demonstrated as a treatment strategy of coronary artery stenosis characterized by long-term safety and efficacy [108]. In recent years, the persistent concern of late stent thrombosis after DES implantation [109,110,111] has led to the development of other lesion treatment strategies such as the DCB to avoid the presence of metallic materials in the coronary vessel [112]. Recently, DCB utilization not only in patients with in-stent restenosis (ISR) but also in patients with de novo small vessel lesion has been proposed, considering the risk of target vessel failure after stent implantation.

The benefit of DCB for treatment of in-stent restenosis (ISR) has been evaluated in several studies and the use of DCB seems particularly suitable in coronary vessels with multiple previous stent layers [113]. Therefore, both DES and DCB for the treatment of ISR are recommended by the European Guidelines on myocardial revascularization with a Ia class of evidence [2]. However, a recent meta-analysis showed that coronary angioplasty with DCB of ISR was less effective than stenting with DES in terms of target lesion revascularization [114]. Given this result, PCI with DCB seems appropriate only in cases of multiple in-stent restenosis where adding other stents layers might represent more risk of restenosis than an effective benefit.

At present, the best treatment strategy of de novo small vessel lesions is still a matter of debate. Indeed, DES implantation in small coronary vessels, determining the component of late lumen loss, may lead to higher rates of ISR [115]. Randomized trials and a meta-analysis compared small vessels treatment between DCB and DES showing conflicting results [113]. Moreover, the definition of small vessels in terms of vessel diameter was not standardized. A previous meta-analysis proved that DES were associated with a significant reduction in percent diameter stenosis compared with DCB [116], while a more recent meta-analysis showed that DCB were noninferior to DES in all clinical outcomes [117]. Randomized trials with a standard definition of small coronary vessels comparing the clinical outcome of patients treated with DES, DCB, and medical therapy alone should clarify first the clinical benefits of small vessels revascularization and second the best treatment strategy. Probably, the most recent techniques of PCI optimization may reduce the rate of target vessel failure also in small vessel disease treated with DES, highlighting the benefit of stent implantation in terms of clinical outcome. Finally, very recent registries showed promising results in terms of the safety, feasibility, and clinical outcomes of the novel sirolimus-coated balloon for coronary stenosis treatment [118,119]. Further investigations are needed to define the clinical implication of this novel DCB in the field of percutaneous revascularization.

## 6. Conclusions

Chronic coronary syndromes include most patients with coronary artery disease. The management of CCS includes prevention, optimal risk factor control, lifestyle modifications, medical treatment, and invasive revascularization. The current available evidence supports the beneficial effect of revascularization on symptoms relief and the reduction of spontaneous myocardial infarction rate in CCS patients; however, no randomized clinical trial to date has demonstrated a decrease in mortality with elective PCI revascularization. Future guidelines should take into account the results coming from these recent trials but at the same time acknowledge their limitations.

## Data Availability

Not applicable.

## Abbreviations

CABG	coronary artery bypass surgery
CAD	coronary artery disease
CCS	chronic coronary syndromes
CCTA	coronary computed tomography angiography
FFR	fractional flow reserve
LVEF	left ventricular ejection fraction
MACE	major cardiac adverse event
OMT	optimal medical therapy
ORBITA	Objective Randomized Blinded Investigation with optimal medical Therapy or Angioplasty in stable angina
PCI	percutaneous coronary intervention
